# Change in product selectivity during the production of glyceric acid from glycerol by *Gluconobacter* strains in the presence of methanol

**DOI:** 10.1186/2191-0855-3-20

**Published:** 2013-04-02

**Authors:** Shun Sato, Naoki Morita, Dai Kitamoto, Toshiharu Yakushi, Kazunobu Matsushita, Hiroshi Habe

**Affiliations:** 1 Research Institute for Innovation in Sustainable Chemistry, National Institute of Advanced Industrial Science and Technology (AIST), Tsukuba Central 5-2, 1-1-1 Higashi, Tsukuba, Ibaraki, 305-8565, Japan; 2 Bioproduction Research Institute, National Institute of Advanced Industrial Science and Technology (AIST), 2-17-2-1 Tsukisamu-higashi, Toyohira-ku, Sapporo, 062-8517, Japan; 3 Department of Biological Chemistry, Faculty of Agriculture, Yamaguchi University, 1677-1 Yoshida, Yamaguchi, 753-8515, Japan

**Keywords:** Acetic acid bacteria, Glyceric acid, Methanol, Membrane-bound alcohol dehydrogenase, Raw glycerol

## Abstract

To enhance the value-added use of methanol-containing raw glycerol derived from biodiesel fuel production, the effect of methanol supplementation on glyceric acid (GA) production by *Gluconobacter* spp. was investigated. We first conducted fed-batch fermentation with *Gluconobacter frateurii* NBRC103465 using raw glycerol as a feeding solution. GA productivity decreased with increasing dihydroxyacetone (DHA) formation when the raw glycerol contained methanol. The results of this experiment and comparative experiments using a synthetic solution modeled after the raw glycerol indicate that the presence of methanol caused a change in the concentrations of GA and DHA, two glycerol derivatives produced during fermentation. Other *Gluconobacter* spp. also decreased GA production in the presence of 1% (*v*/*v*) methanol. In addition, purified membrane-bound alcohol dehydrogenase (mADH) from *Gluconobacter oxydans*, which is a key enzyme in GA production, showed a decrease in dehydrogenase activity toward glycerol as the methanol concentration increased. These results strongly suggest that the observed decrease in GA production by *Gluconobacter* spp. resulted from the methanol-induced inhibition of mADH-mediated glycerol oxidation.

## Introduction

Biodiesel fuel (BDF), a class of renewable energy, is widely used to conserve fossil fuels and reduce carbon dioxide emissions. One method of BDF production involves transesterification between triacylglycerols, which are present in plant oils and animal fats, and methanol under alkaline conditions. This reaction forms glycerol as a byproduct at 10% of the initial amount of triacylglycerol. Because BDF production has rapidly increased in the US and in European and Asian countries (Rahmat et al. 2010; Glycerin market report, 2011), residual glycerol waste (raw glycerol) is now being used in chemical industries as an alternative feedstock. Therefore, many research groups have developed chemical and biological techniques for the conversion of glycerol into value-added chemicals such as epichlorohydrine (Kubicek et al. 2005), 1,3-propanediol (Rehman et al. 2008), 3-hydroxypropionic acid (Rathnasingh et al. 2009), and 2,3-dihydroxypropionic acid (glyceric acid, GA) (Habe et al. 2009a).

Raw glycerol derived from BDF production has diverse properties that depend on the initial raw materials (e.g., origin of triacylglycerol), reaction conditions, and manufacturing process. The transesterification of triacylglycerol with methanol requires excess methanol for efficient BDF production, resulting in an impure, methanol-containing raw glycerol. Although methanol recovery by evaporation during the BDF manufacturing process is relatively easy, it requires additional energy and is costly. Therefore, the use of raw glycerol is economically preferable, and so the technological utility of glycerol that contains impurities, such as methanol and alkali metals, should be developed. Indeed, raw glycerol after purification has relatively high purity and represents a useful raw material for chemical production, whereas impure raw glycerol is often wasted. Therefore, it is important to investigate the effects of impurities in raw glycerol, particularly methanol, on chemical production.

Our recent research has focused on microbial GA production from glycerol and the applications of GA because of its simple but chiral structure, which provides building blocks for various fine chemicals (Habe et al. 2009b). GA itself has been reported to have biological activity, such as accelerating the oxidation of ethanol (Eriksson et al. 2007) and enhancing the viability of ethanol-dosed gastric cells (Habe et al. 2011a). Applications of GA as a value-added material, including as an antitrypsin compound (Habe et al. 2011b), a bioplastic monomer (Fukuoka et al. 2011), and novel surfactants (Fukuoka et al. 2012), have also been investigated. With regard to the production of GA, a biological method that involves the enantioselective conversion of glycerol to D-GA by acetic acid bacterial fermentation has been developed (Habe et al. 2009c). *Gluconobacter frateurii* NBRC103465 showed the highest GA productivity (136 g/L), whereas *Acetobacter tropicalis* NBRC16470 produced D-GA with 99% ee (Habe et al. 2009a).

Concerning the effect of raw glycerol containing methanol on GA production by acetic acid bacteria, we demonstrated that *Gluconobacter* sp. NBRC3259 produced less GA when raw glycerol from which impurities had not been removed was applied (Habe et al. 2009d). It was also shown that this strain produced only a small amount of GA in the presence of 1% (*v*/*v*) methanol. In contrast, we developed a fed-batch fermentation method that uses glycerol in an alkaline solution for glycerol feeding and pH control (Habe et al. 2009a). Because raw glycerol usually contains alkaline metals such as sodium and potassium, it is of interest to investigate the feasibility of utilizing raw glycerol in fed-batch fermentation.

**Figure 1:**
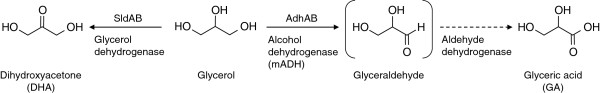
**Pathways for GA and DHA formation from glycerol by *Gluconobacter *spp. **The dashed arrow indicates an unidentified enzymatic reaction in GA formation. AdhAB, membrane-bound alcohol dehydrogenase, which is referred to as mADH; SldAB, membrane-bound glycerol dehydrogenase, which is referred to as SLDH or GLDH.

In this study, we found that GA production decreased, whereas the production of dihydroxyacetone (DHA), a byproduct of GA fermentation (Figure 1), increased when we used raw glycerol as a feeding solution for fed-batch fermentation with *G. frateurii* NBRC103465. Hence, to elucidate the reason for the change in the glycerol derivatives produced, we investigated GA production by various *Gluconobacter* spp. and glycerol oxidation in the presence of methanol.

## Materials and methods

### Bacterial strains and culture conditions

*Gluconobacter frateurii* NBRC103465, *G. frateurii* THD32 (Toyama et al. 2005) and its *ΔsldA* mutant (Toyama et al. 2005; Soemphol et al. 2008), and *Gluconobacter oxydans* IFO12528 and its *ΔadhA* mutant (Habe et al. 2009a) were used for GA production. Seed cultures of the *Gluconobacter* strains were prepared in 5 mL of glucose medium composed of 5 g/L glucose, 5 g/L yeast extract, 5 g/L polypepton, and 1 g/L MgSO_4_ · 7H_2_O at 30°C and 200 rpm for 24 h. The seed cultures (1.5 mL) were transferred to 300-mL Erlenmeyer flaks containing 30 mL of glycerol medium composed of 170 g/L glycerol, 10 g/L polypepton, 1 g/L yeast extract, 1 g/L MgSO_4_ · 7H_2_O, 0.9 g/L KH_2_PO_4_, and 0.1 g/L K_2_HPO_4_. The cultures were incubated at 30°C and 200 rpm on a rotary shaker for 96 h. When needed, 50 mg/L kanamycin for the *ΔadhA* strain of *G. oxydans* and the *ΔsldA* strain of *G. frateurii* THD32 was added to the medium.

### Jar fermentation experiment

*Gluconobacter frateurii* was cultured in a 5-L jar fermenter containing 2.5 L of glycerol medium (Habe et al. 2009a). The seed cultures (90 mL) were used for inoculation. Cultivation was done at 30°C, 500 rpm, and 0.5 vvm, and maintained at pH 6 using raw glycerol derived from BDF production or the model solution. The raw glycerol contained the following components: glycerol, 66.4% (*w*/*v*); methanol, 30.9% (*w*/*v*); and sodium salt, 0.54% (*w*/*v*); pH 12 (Habe et al. 2009d). In contrast, based on the raw glycerol composition, a synthetic solution modeled after the raw glycerol was prepared with the following pure reagents: glycerol, 66% (*w*/*v*); methanol, 30% (*w*/*v*); and sodium hydroxide, 1 M.

### Analysis of the culture broth

The glycerol, GA, and DHA concentrations in the culture broth were determined by high-performance liquid chromatography (HPLC), as described previously (Habe et al. 2009a). In addition, the methanol concentration was determined by HPLC using the same method as that employed for the GA assay.

Bacterial growth was evaluated by OD measurements at 600 nm using a V-530 UV/VIS spectrophotometer (JASCO Corp., Tokyo, Japan).

### Evaluation of the ability of *G. oxydans* and its *ΔadhA* mutant to assimilate methanol

Whole cells of *G. oxydans* IFO12528 and its *ΔadhA* strain were used to investigate methanol consumption. Cells were precultured in 30 mL of glucose medium in 300-mL Erlenmeyer flasks at 30°C and 200 rpm for 24 h. Cells collected from 60 mL of the culture by centrifugation were suspended in 30 mL of glycerol medium containing 0–10% (*v*/*v*) methanol. The initial OD_600_ was ~2.0. The flasks were shaken at 30°C and 200 rpm, and an aliquot of the broth was removed at regular intervals for HPLC.

### Enzyme assays

Glycerol dehydrogenase activity in the presence of methanol was evaluated using purified, membrane-bound *G. oxydans* alcohol dehydrogenase (mADH) based on the method of Adachi et al. (1978) with modifications. Briefly, the reaction mixture contained 0.4 mL of McIlvaine buffer (pH 5.0), 10 mM potassium ferricyanide, 8 mM sodium azide, enzyme solution, 5-20% (*w*/*v*) glycerol, and/or 0-2% (*w*/*v*) methanol in a total volume of 1.0 mL. The reaction was carried out at 25°C for 5 min by adding ferricyanide solution, and stopped by adding 0.5 mL of ferric sulfate-Dupanol reagent (Wood et al. 1962). The resulting mixture was allowed to stand at 25°C for 20 min, and then the absorbance of the solution was measured at 660 nm to estimate the intensity of the Prussian blue color formed. The oxidation of 1 μmol of substrate was equal to 4.0 absorbance units. One unit of dehydrogenase activity was defined as the amount of enzyme that catalyzed the oxidation of 1 μmol of substrate in 1 min. After the determination of dehydrogenase activity toward 1.2 and 2% (*w*/*v*) methanol, that towards 20% (*w*/*v*) glycerol in the presence of various methanol concentrations was calculated by subtracting the value of the activity toward methanol from that toward glycerol in the presence of methanol.

The protein concentration of the purified enzyme was determined by the Lowry method using bovine serum albumin as the standard.

## Results

### Fed-batch GA fermentation by *G. frateurii* NBRC103465 using raw glycerol derived from BDF production and its modeled solution

**Figure 2:**
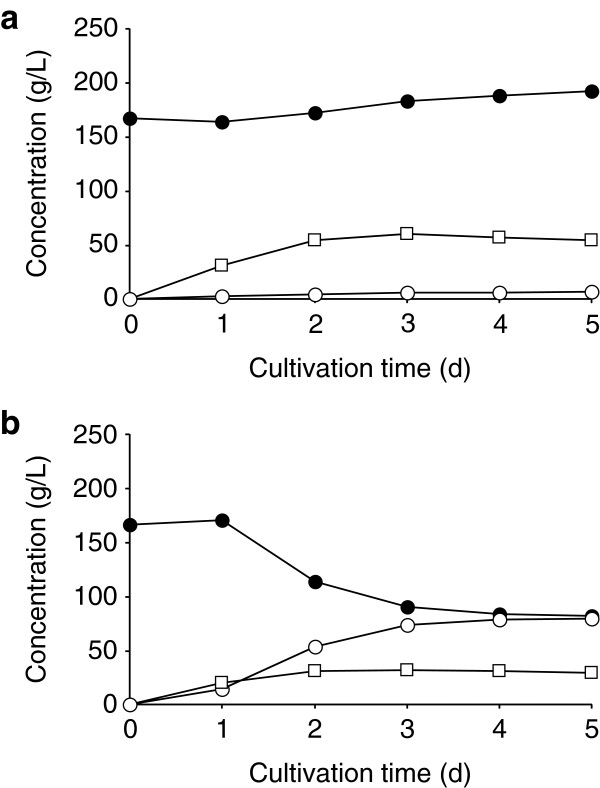
**Time-dependent changes in the concentrations of GA, DHA, and glycerol in *G. frateurii *NBRC103465 cultures. **Cells were cultivated in a 5-L jar fermenter containing 2.5 L of glycerol medium at 30°C, 500 rpm, and 0.5 vvm. The pH of the cultures was controlled using BDF production-derived raw glycerol containing 30.9% (*w*/*v*) methanol (**a**); the control contained no methanol (**b**). Open circles: GA concentration; open squares: DHA concentration; closed circles: glycerol concentration.

Previously, Habe et al. (2009a) demonstrated that fed-batch fermentation using glycerol in an alkaline feeding solution resulted in considerable improvement in GA production by *G. frateurii* NBRC103465, indicating a potential use for raw glycerol derived from BDF production, which contains both alkali metals and methanol. Therefore, we first attempted to use raw glycerol (66.4% *w*/*v* glycerol and 30.9% *w*/*v* methanol, pH 12; Habe et al. 2009d) as a feeding solution for GA fermentation by *G. frateurii* NBRC103465. As shown in Figure 2a, GA was barely detectable in the culture broth, whereas DHA was present at >50 g/L after 5 days of cultivation. In contrast, after the removal of methanol (40% *w*/*v* glycerol), 81 g/L GA and 38 g/L DHA were produced after 5 days of cultivation, despite the presence of other impurities in the original glycerol (Figure 2b). This suggests that the addition of methanol along with raw glycerol to the culture altered the production of GA and DHA by *G. frateurii*. Note that glycerol content in the culture using raw glycerol containing methanol as a feeding solution slightly increased during the cultivation, though 51 g/L of DHA was produced (Figure 2a). In this case, the feeding rate of glycerol accompanied with pH control exceeded the consumption rate of glycerol for cell growth and DHA production, resulting in a gradual accumulation of glycerol in the culture broth. In contrast, glycerol content in the culture using raw glycerol containing no methanol as a feeding solution decreased during the cultivation (Figure 2b), probably because a large amount of GA was produced.

**Figure 3:**
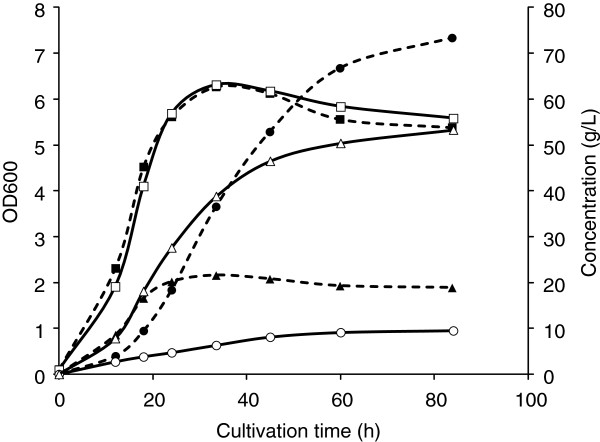
**Time-dependent changes in cell growth and the concentrations of GA and DHA in *G. frateurii *NBRC103465 cultures. **Cells were cultivated in a 5-L jar fermenter containing 2.5 L of glycerol medium at 30°C, 500 rpm, and 0.5 vvm. The pH of the cultures was controlled using a synthetic solution modeled after the raw glycerol, which contains 30% (*w*/*v*) methanol (solid lines, open symbols); the control contained no methanol (broken lines, closed symbols). Cell growth was evaluated by measurement of the OD at 600 nm. Squares: cell growth; circles: GA concentration; triangles: DHA concentration.

To clarify whether the above change in product selectivity was caused by methanol supplementation or by that in combination with other impurities in the raw glycerol (e.g., free fatty acids, methyl esters of fatty acids, and residual triacylglycerols), the same experiment was performed using a synthetic solution modeled after the raw glycerol. This solution consisted of 66% (*w*/*v*) glycerol and 30% (*w*/*v*) methanol in 1 M NaOH; a methanol-free version of the solution was used as a control. Figure 3 shows the cell growth and GA and DHA production profiles of *G. frateurii*. Cell growth was unaffected, but GA and DHA production were altered by methanol supplementation. In the absence of methanol, GA predominated (73 g/L); only 20 g/L DHA had been produced after 84 h of cultivation. In contrast, in the presence of methanol, DHA predominated (>50 g/L) and the GA level was <10 g/L. This result shows that methanol caused a change in product selectivity between GA and DHA with almost the same bacterial growth profile.

### GA production by *Gluconobacter* spp. in the presence of methanol

To determine the effect of methanol on GA production by *Gluconobacter* strains other than *G. frateurii* NBRC103465, we examined GA and DHA production from glycerol in the presence of methanol by *G. frateurii* THD32 and *G. oxydans* IFO12528. The strains were cultivated in 300-mL flasks containing 30 mL of glycerol medium. Table 1 summarizes the amounts of GA and DHA produced after 96 h of cultivation. All of the strains tested produced less GA in the presence of 1% (*v*/*v*) methanol than with no methanol. In addition, the amount of DHA produced was increased in methanol-supplemented cultures of *G. frateurii* NBRC103465 and *G. oxydans* IFO12528, whereas methanol supplementation did not affect DHA production in the *G. frateurii* THD32 cultures. This result suggests that methanol decreases GA production by *Gluconobacter* spp.

**Table 1:** **Effect of methanol supplementation on GA and DHA production by *Gluconobacter *spp.**

	***G. frateurii *NBRC103465**		***G. frateurii *THD32**		***G. oxydans *IFO12528**	
Initial methanol (%, *v*/*v*)	0	1	0	1	0	1
GA (g/L)	28.2 ± 0.3	14.4 ± 0.7	23.0 ± 0.3	6.8 ± 0.3	18.8 ± 1.0	5.9 ± 0.4
DHA (g/L)	15.9 ± 0.6	38.0 ± 1.7	25.1 ± 5.8	24.1 ± 1.4	33.7 ± 0.3	51.9 ± 2.1

Cells were cultivated in 30 mL of glycerol medium containing 170 g/L glycerol with methanol supplementation at 30°C for 96 h. The averages and standard deviations of two independent experiments are presented.

### Effect of methanol supplementation on the *G. oxydans* Δ*adhA* mutant and purified mADH

**Figure 4:**
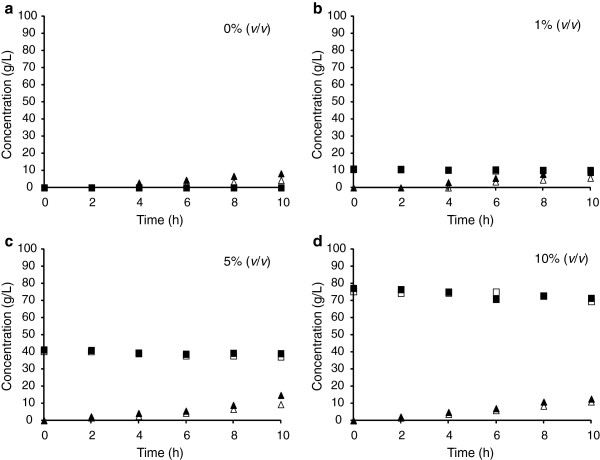
**Time-dependent changes in the concentrations of methanol and DHA in cultures of *G. oxydans *and its *ΔadhA *mutant. **Cells precultured in glucose medium for 24 h were inoculated into glycerol medium with 0 (**a**), 1 (**b**), 5 (**c**), or 10% (*v*/*v*) (**d**) methanol at an OD_600 _of ~2.0 and incubated at 30°C and 200 rpm. The averages from two independent experiments are presented. Open symbols, *G. oxydan*s IFO12528; closed symbols, *G. oxydans ΔadhA* mutant.

We investigated the effect of methanol on the activity of a key enzyme in GA production, mADH, which catalyzes the oxidation of glycerol to glyceraldehyde and is encoded by *adhA* (Habe et al. 2009a, 2010). This enzyme exhibits weak methanol dehydrogenase activity as compared to ethanol (Adachi et al. 1978). Therefore, we first investigated whether methanol was consumed by mADH using both the parental and *adhA*-deficient mutant (*ΔadhA*) strains of *G. oxydans*. Both strains were cultured in glucose medium for 24 h and then inoculated into glycerol medium containing up to 10% (*v*/*v*) methanol. As shown in Figure 4, no significant differences in methanol concentration in the culture broth were observed between the parental and *ΔadhA* strains of *G. oxydans* for up to 10 h, indicating that mADH is not directly involved in methanol oxidation. In addition, methanol supplementation to the *ΔadhA* strain did not significantly alter the production of DHA (Figure 4).

**Figure 5:**
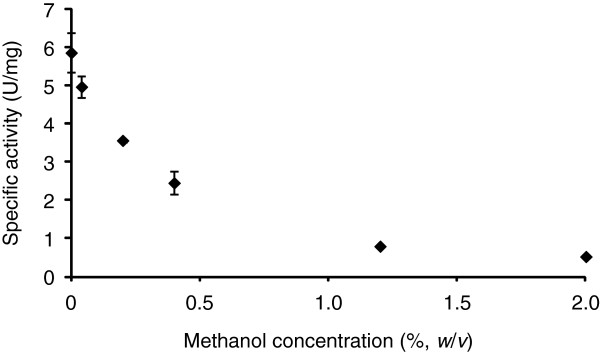
**Methanol inhibition of glycerol oxidation by mADH. **The specific activity toward 20% (*w*/*v*) glycerol was measured using potassium ferricyanide as the electron acceptor. The data are presented as the averages and standard deviations from three independent experiments.

Next, we measured glycerol dehydrogenase activity in the presence of methanol using purified mADH. Glycerol dehydrogenase activity in the absence of methanol increased as the glycerol concentration increased, reaching 5.9 U/mg at 20% (*w/v*) glycerol. At this concentration, a rapid decrease in dehydrogenase activity with increasing methanol concentration was observed (Figure 5). In the presence of 2% (*w/v*) methanol, glycerol dehydrogenase activity decreased to one tenth that in the absence of methanol. The half-maximal inhibitory concentration (IC_50_) of methanol was estimated to be ~0.31% (*w*/*v*). These results suggest that <1% (*w*/*v*) methanol inhibited mADH-catalyzed oxidation at a high concentration of glycerol.

## Discussion

Our data indicate that GA production by *G. frateurii* NBRC103465 decreased when the microbe was cultured using raw glycerol (Figure 2) and a synthetic solution modeled after the raw glycerol, which contains 30% (*w*/*v*) methanol (Figure 3), for pH control. This phenomenon also occurred in flask cultures of other *Gluconobacter* spp. (Table 1). Thus, methanol was likely the cause of the decrease in GA production.

Previously, we showed that mADH is a key enzyme in the production of GA from glycerol, and that its deletion from *G. oxydans* (***Δ****adhA*) resulted in a strain incapable of producing GA (Habe et al. 2009a). In addition, mADH exhibits higher dehydrogenase activity toward methanol than toward glycerol (Adachi et al. 1978). Therefore, we hypothesized that mADH catalyzed the oxidation of methanol more easily than the oxidation of glycerol, resulting in a decrease in the methanol consumption rate in the broth of the *ΔadhA* mutant. However, no differences in methanol consumption between the parental and *ΔadhA* strains of *G. oxydans* grown in glycerol containing up to 10% (*v*/*v*) methanol were detected (Figure 4). Next, the inhibitory effect of methanol on glycerol oxidation by mADH was evaluated using purified mADH. A comparison of mADH activity at high glycerol concentrations with or without methanol demonstrated that methanol inhibited glycerol dehydrogenase activity (Figure 5). This suggests that methanol decreased the rate of glycerol oxidation by mADH, resulting in reduced GA production. Activity of mADH toward various amount of glycerol (5, 10, 15, and 20%, *w*/*v*) in the presence of methanol (0.3%, *w*/*v*) was also measured. A gradual increase in dehydrogenase activity was observed as the glycerol concentration increased, although methanol inhibited dehydrogenase activity constantly by approximately 60% as compared to that in the absence of methanol (data not shown). This could suggest noncompetitive inhibition by methanol in mADH glycerol dehydrogenation; however, detailed kinetic analyses of mADH activity toward glycerol and/or methanol will be necessary to clarify the mechanism whereby methanol inhibits the oxidation of glycerol by mADH. In addition, fed-batch cultivation with a raw glycerol model solution (Figure 3) revealed that the methanol concentration in the broth reached 0.4 and 1.5% after 12 and 18 h of cultivation, respectively (data not shown). Considering the IC_50_ value for methanol in the oxidation of glycerol by mADH, GA production was probably inhibited even in the early stages of cultivation.

Because the Δ*adhA* strain of *G. oxydans* showed similar DHA production profiles in the presence of methanol as well as the parental strain (Figure 4), methanol was supposed to enhance DHA production regardless of GA production. In contrast, our preliminarily experiment showed that a membrane-bound glycerol dehydrogenase (SldAB)-defective mutant of *G. frateurii* THD32 (*ΔsldA*), which is incapable of producing DHA, showed a clear decrease in GA production in the presence of 1% methanol (data not shown), as well as the parental strain *G. frateurii* THD32. This suggests that methanol inhibited GA production regardless of DHA production. These complementary experiments imply that methanol would have an influence on activities of individual enzymes involved in each production. In contrast, DHA production by *G. frateurii* THD32 was not significantly changed in the presence of 1% methanol (Table 1). Hence, studies on cell responses at the transcriptomic and metabolomic levels in the presence of methanol will help elucidate the detailed mechanism of methanol-related DHA production and the difference among *Gluconobacter* strains in effect of methanol on DHA production.

*Gluconobacter* spp. tend to produce both GA and DHA at a high concentration (170 g/L) of glycerol (Table 1). In terms of DHA production, this is a problem; that is, a high concentration of glycerol during DHA fermentation results in increased byproduct (GA) formation (Figure 1). In addition, a higher initial concentration of glycerol decreased the rates of cell growth and DHA production (Claret et al. 1992). Therefore, attempts have been made to improve DHA production by process engineering (Hu et al. 2011) and strain development (Habe et al. 2010). However, our data show that *G. oxydans*, an industrial strain used for DHA production, produced a large quantity of DHA from 170 g/L glycerol with less GA production in the presence of 1% methanol (Table 1). This indicates that efficient DHA production by *G. oxydans* using a high concentration of glycerol can be achieved by adding a small amount of methanol to the culture. In this case, methanol seemed to act not only as an inhibitor of GA formation, but also as an enhancer of DHA formation. This suggests that BDF-derived raw glycerol containing methanol would be a good source material for efficient and economical DHA production with less byproduction of GA, although a clear mechanism for enhancing DHA production is unclear.

In summary, GA production in the presence of methanol decreased with the production of DHA by *Gluconobacter* spp. The dehydrogenase activity of *G. oxydans* mADH toward glycerol was inhibited by methanol, suggesting that the rate of glycerol oxidation catalyzed by mADH determines the product selectivity of *Gluconobacter* spp. between GA and DHA. Recombinant *G. frateurii* with an enhanced ability to assimilate methanol or mADHs with a lower affinity for methanol will be necessary for the efficient production of GA from raw glycerol.

## Competing interests

The authors declare that they have no competing interests.

## Authors’ contributions

HH, TY, NM, DK, and KM designed research; SS, HH, and TY performed research; SS, HH, and TY analyzed data; and SS and HH wrote the paper. All authors read and approved the final manuscript.
